# Supplementary material for: Crystal structure of human Mediator subunit MED23
Source: Nat Commun. 2018 Aug 23;9:3389. doi: 10.1038/s41467-018-05967-y (PMC6107663; doi:10.1038/s41467-018-05967-y)
Supplement: Supplementary file 1 — Supplementary Information [file 41467_2018_5967_MOESM1_ESM.pdf]

Supplementary Information

Monté et al., Crystal structure of Human Mediator subunit MED23

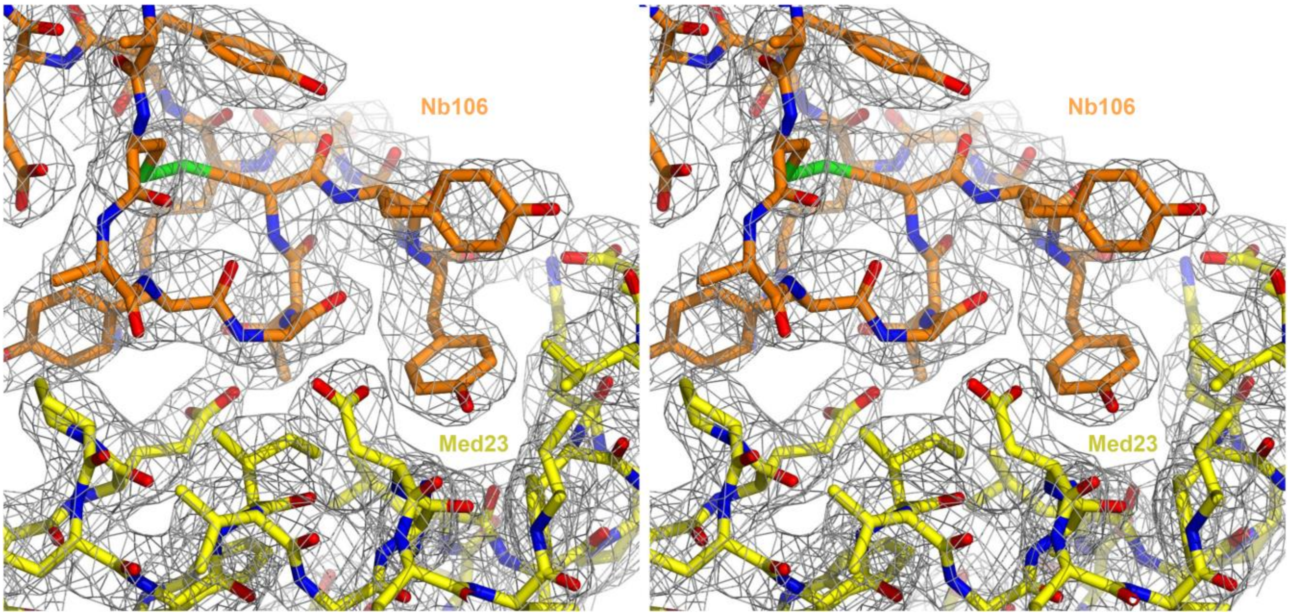

Supplementary Figure 1 Stereo view of the 2Fo-Fc electron density map contoured at 1.2σ showing the interface between the Nb106 nanobody and MED23. Carbon atoms of Nb106 and MED23 are in orange and yellow, respectively.

```
        H1  HR1  H2  H3  H4  HR2  H5
1       METQLQSIFEEVVKTEVIEEAFPGMFMDTPEDEKTKLISCLGAFRQFWGGLSQESHEQCIQWIVKFIHGQHSPKRISFLY

        H5  H6  HR3  H7  H8  HR4  H9
81      DCLAMAVETGLLPPRLVCESLINSDTLEWERTQLWALTFKLVRKIIGGVDYKGVRDLLKVILEKILTIPNTVSSAVVQQL

        H9  H10  H11  HR5  H12  H13  H14
161     LAAREVIAYILERNACLLPAYFAVTEIRKLYPEGKLPHWLLGNLVSDFVDTFRPTARINSICGRCSLLPVVNNSGAICNS

        H15  H16  HR1  H17  H18
241     WKLDPATLRFPLKGLLPYDKDLFEPQTALLRYVLEQPYSRDMVCNMLGLNKQHKQRCPVLEDQLVDLVVYAMERSETEEK

        HR2  H19  H20  HR3  H21  H22
321     FDDGGTSQLLWQHLSSQLIFFVLFQFASFPHMVLSLHQKLAGRGLIKGRDHLMWVLLQFISGSIQKNALADFLPVMKLFD

        H23  H24  H25
401     LLYPEKEYIPVPDINKPQSTHAFAMTCIWIHLNRKAQNDNSKLQIPIPHSLRLHHEFLQQSLRNKSLQMNDYKIALLCNA

        H26  H27  H28  HR1
481     YSTNSECFTLPMGALVETIYGNGIMRIPLPGTNCMASGSITPLPMNLLDSLTVHAKMSLIHSIATRVIKLAHAKSSVALA

        H29  H30  HR2  H31  H32  HR3
561     PALVETYSRLLVYMEIESLGIKGFISQLLPTVFKSHAWGILHTLLEMFSYRMHHIQPHYRVQLLSHLHTLAAVAQTNQNQ

        H33  H34  H35  HR4  H36
641     LHLCVESTALRLITALGSSEVQPQFTRFLSDPKTVLSAESEELNRALILTLARATHVTDFFTGSDSIQGTWCKDILQTIM

        H37  HR5  H38  H39  HR1  H40  H41
721     SFTPHNWASHTLSCFPGPLQAFFKQNNVPQESRFNLKKNVEEEYRKWKSMSNENDIITHFSMQGSPPLFLCLLWKMLLET

        HR2  H42  H43  HR3  H44  H45  HR4
800     DHINQIGYRVLERIGARALVAHVRTFADFLVYEFSTSAGGQQLNKCIEILNDMVWKYNIVTLDRLILCLAMRSHEGNEAQ

        H46  H47  HRins  H48  H49  H50
881     VCYFIIQLLLLKPNDFRNRVSDFVKENSPEHWLQNDWHTKHMNYHKKYPEKLYFEGLAEQVDPPVQIQSPYLPIYFGNVC

        H51  HR5  H52  H53  HR6  H54
961     LRFLPVFDIVIHRFLELLPVSKSLETLLDHLGGLYKFHDRPVTYLYNTLHYYEMHLRDRAFLKRKLVHAIIGSLKDNRPQ

        H55  H56  HR1  H57  H58
1041    GWCLSDTYLKCAMNAREENPWVPDDTYYCRLIGRLVDTMAGKSPGPFPNCDWRFNEFPNPAAHALHVTCVELMALAVSGK

        H59  HR2  H60  H61  H62  HR3  H63
1121    EVGNALLNVVLKSQPLVPRENITAWMNAIGLIITALPEPYWIVLHDRIVSVISSPSLTSETEWVGYPFRLFDFTACHQSY

        H64  H65  HR4  H66  H67  H68
1201    SEMSCSYTLALAHAVWHHSSIGQLSLIPKFLTEVLLPIVKTEFQLLYVYHLVGPFLQRFQQERTRCMIEIGVAFYDMLLN

        HR5  H69  H70  HR6  H71
1281    VDQCSTHLNYMDPICDFLYHMKYMFTGDSVKEQVEKIICNLKPALKLRLRFITHISKMEPAAVPPQAMNSGSPAPQSNQV

1361    PVSLPVTQ
```

Supplementary Figure 2 Amino acid sequence of MED23 and definition of structural elements. α–helices are boxed in grey; 3_{10} elements are boxed in pink. Unstructured regions not visible in the electron density are boxed in orange. The two small strands in loop H26-H27 are boxed in blue. R611 is highlighted in red. The heat repeat (HR) motifs for each solenoid domain are shown in orange (N-HEAT,), in yellow (3-HEAT), in red (5-HEAT), in green (6-HEAT) and in blue (C-HEAT), with numbering of helices. Secondary structures are defined using STRIDE[1] as implemented in VMD[2].

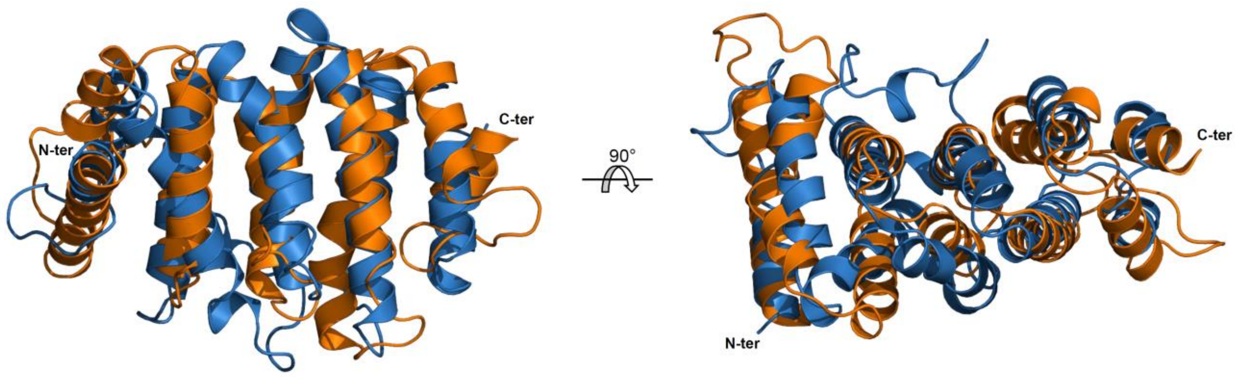

Supplementary Figure 3 Superposition of N-HEAT (heat repeat motifs 1-5, in orange) and C-HEAT (heat repeat motifs 21-25, in blue). The two α-solenoids share the same organization, which is reminiscent of MIF4G-like domains. The Cα of the heat repeat motifs superimpose with an rmsd of 3.3Å.

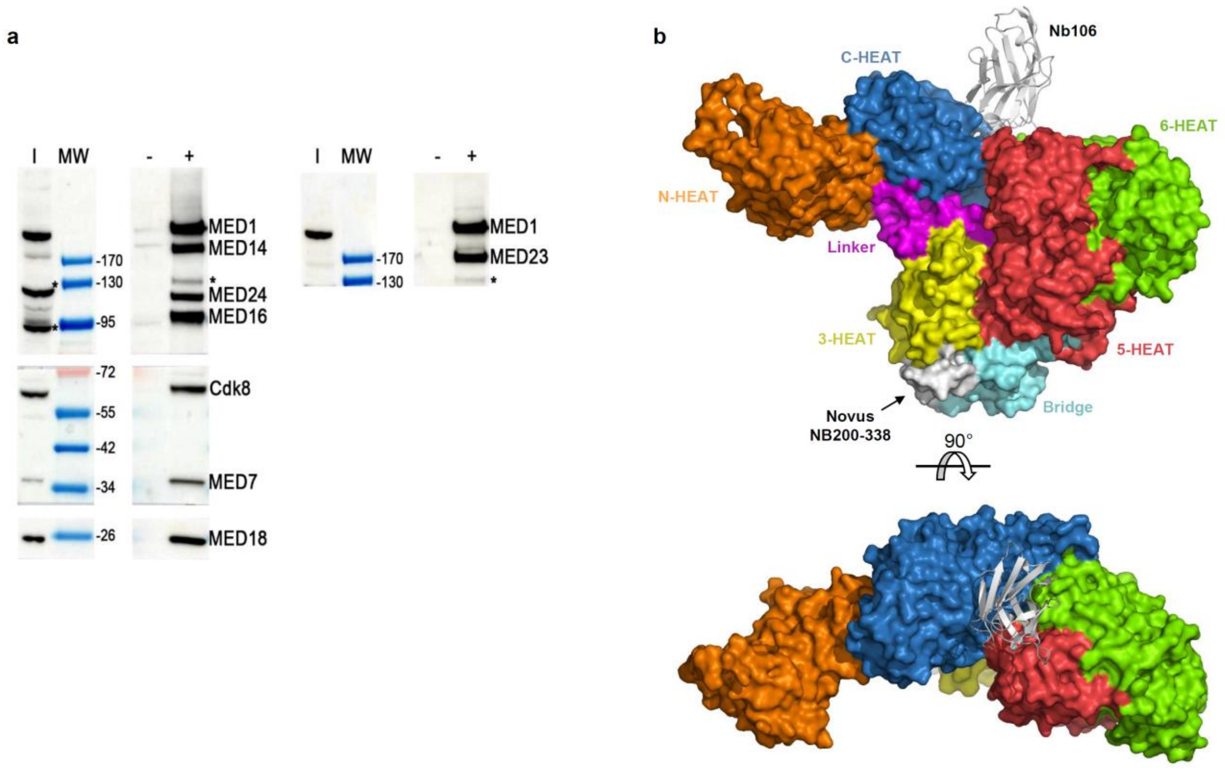

Supplementary Figure 4

a. Co-immunoprecipitation of Mediator using Nb106. To check if Nb106 also interacts with MED23 within the Mediator complex in a cellular context, co-immunoprecipation experiments were done on HEK-293 total cellular extracts. When MED23 was recovered with Nb106, subunits encompassing all Mediator modules were also pull-downed. These results clearly indicate that Nb106 interacts with MED23 incorporated within the Mediator complex. I: Input 1/100; MW: Molecular Weight Marker. Size (170, 130, 95, 72, 55, 42, 34 and 26 kDa) are indicated on the right size; -: negative control CaptureSelect affinity matrix; +: CaptureSelect affinity matrix + Nb106. The same blot was successively hybridized with different primary antibodies combinations: (upper panel) Med1-Med23-Med16-Med24, Med14, (middle panel) Cdk8-Med7 and (lower panel) Med18. The * represents unknown bands.

b. Representation of MED23 in complex with nanobody Nb106. The surface of MED23 is represented, with domains colour-coded as in Fig.1. Nb106 is represented as a cartoon, coloured in white. Nb106 binds at an interface between 5-HEAT, 6-HEAT and C-HEAT on one side of the MED23 arch. The region of MED23 targeted by a commercial antibody that co-immunoprecipitates the Mediator complex and targets region 400-450 within the bridge domain is shown coloured in white and is indicated (Novus, NB200-338).

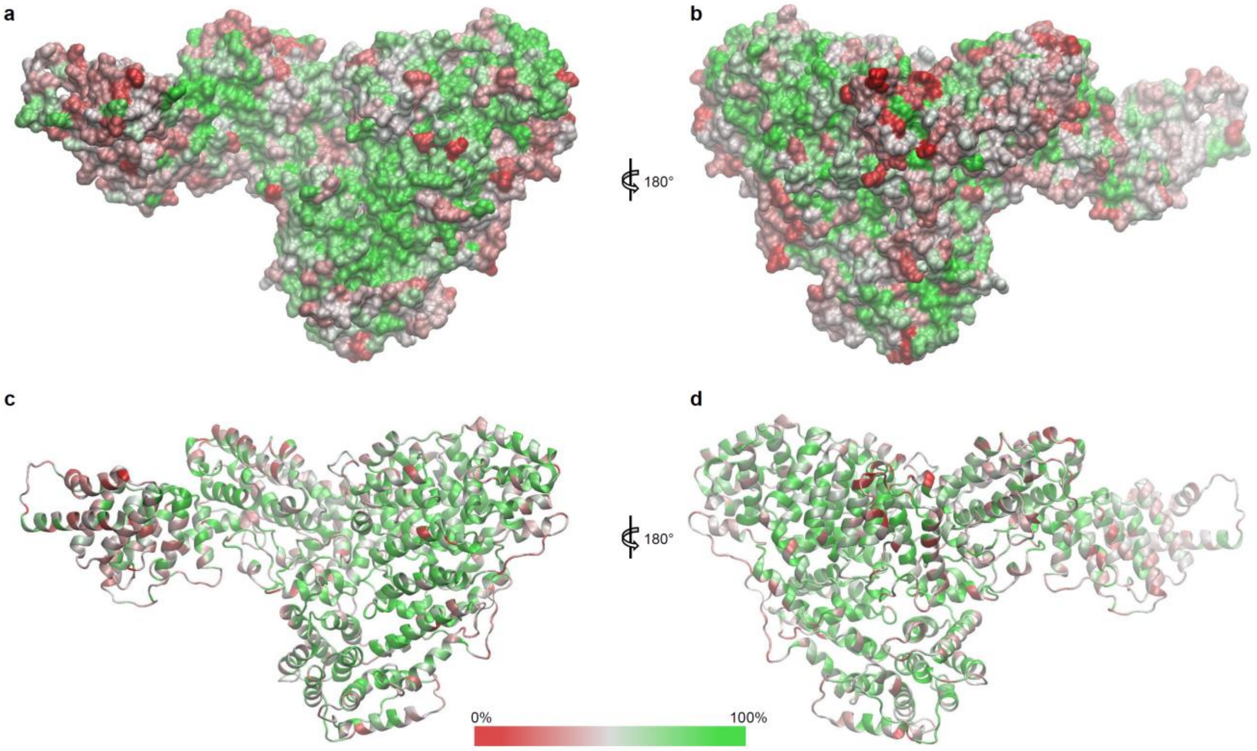

Supplementary Figure 5 Evolutionary analysis of MED23. The human MED23 model is shown both in sphere (**a, b**) and in ribbon representations (**c, d**) colored on the basis of sequence conservation across 19 metazoan species (*Homo sapiens sapiens*, *Mus musculus*, *Gallus gallus*, *Xenopus tropicalis*, *Danio rerio*, *Tetraodon nigroviridis*, *Fugu rubripes/Takifugu rubripes*, *Ciona intestinalis*, *Ciona savignyi*, *Drosophila melanogaster*, *Drosophila pseudoobscura*, *Anopheles gambiae*, *Aedes egypti*, *Apis mellifera*, *Bombyx mori*, *Tribolium casteneum*, *Caenorhabditis elegans*, *Caenorhabditis briggsae*, *Brugia malayi*). The sequence alignment used to prepare the figure has been generated with ClustalW and the representation made with MultiSeq[3], as implemented in VMD[2]. The views illustrate the concave (**a, c**) and convex (**b, d**) faces of MED23, respectively.

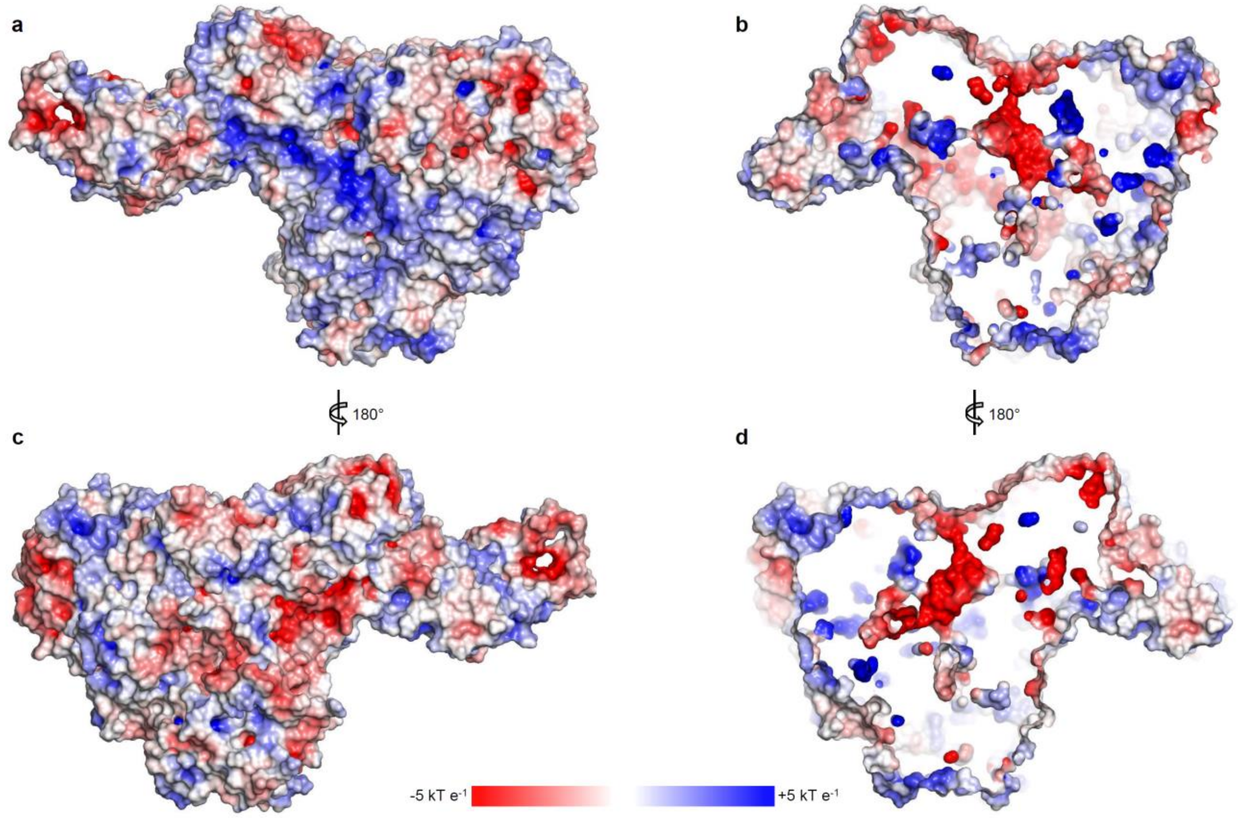

Supplementary Figure 6 Electrostatics analyses of MED23. The surfaces of the concave (**a**) and convex (**b**) faces of MED23 are shown. **c, d** Sectional views of MED23 illustrating the presence of a buried negatively charged cavity within core MED23, at the interface between solenoid domains. Analyses have been performed with APBS (Adaptative Poisson-Bolzmann Solver program)[4] and rendered using PyMOL[5].

	Native $_{monoclinic}$	Native $_{orthorhombic}$	Pt derivative	Au derivative
**Data collection**				
Beamline (synchrotron)	PROXIMA-2A (SOLEIL)	ID29 (ESRF)	ID29 (ESRF)	ID23-1 (ESRF)
Space group	$P2_1$	$P2_12_12_1$	$P2_12_12_1$	$P2_12_12_1$
a, b, c (Å)	75.65, 137.18 90.11	76.15, 119.07, 213.45	75.54, 118.55, 213.50	76.83, 115.77, 222.54
α, β, γ (°)	90, 110.98, 90	90, 90, 90	90, 90, 90	90, 90, 90
Wavelength (Å)	0.98011	1.07166	1.07166	1.03904
Resolution (Å)	49.21-2.65 (2.70-2.65)	48.70-3.00 (3.10-3.00)	48.67-3.75 (3.80-3.75)	48.47-3.85 (3.90-3.85)
Rmerge	0.147 (2.259)	0.139 (2.538)	0.228 (1.637)	0.134 (1.399)
Rmeas	0.155 (2.368)	0.145 (2.635)	0.246 (1.764)	0.160 (1.678)
I/σ(I)	14.26 (1.18)	14.39 (1.14)	6.59 (1.02)	6.51 (1.05)
$CC_{1/2}$	99.9 (66.6)	99.9 (46.6)	99.5 (52.2)	99.5 (36.2)
Completeness (%)	99.8 (99.8)	100 (100)	100 (100)	99.5 (99.7)
Redundancy	21.1 (22.0)	13.4 (13.9)	7.1 (7.2)	3.4 (3.3)
**Refinement**				
Resolution (Å)	15.0 – 2.80 (2.90 – 2.80)			
No . unique reflections	42643 (4250)			
I/σ(I)	16.2 (2.15)			
R_{work} / R_{free}	0.199 (0.240)			
No. Atoms<br>Protein	 11710			
B-factors<br>Protein	 91.5			
R.m.s. deviations<br>Bond lengths(Å)<br>Bond angles (°)	 0.004<br>0.92			

Supplementary Table 1 Data collection and refinement statistics. Values in parentheses are for highest resolution shell. Only one crystal was used to measure each diffraction data set. Rmeas is defined as followed: $R_{meas} = \Sigma_{hkl} \{N(hkl)/[N(hkl)\text{-}1\}^{1/2} \Sigma_i |I_i(hkl) - (I(hkl))|/ \Sigma_{hkl} \Sigma_i I_i(hkl)$, where N is the number of times a given reflection has been observed.

Supplementary References

1. Frishman D, Argos P. Knowledge-based protein secondary structure assignment. *Proteins* **23**, 566-579 (1995).

2. Humphrey W, Dalke A, Schulten K. VMD: visual molecular dynamics. *J Mol Graph* **14**, 33-38 (1996).

3. Roberts E, Eargle J, Wright D, Luthey-Schulten Z. MultiSeq: unifying sequence and structure data for evolutionary analysis. *BMC Bioinformatics* **7**, 382 (2006).

4. Jurrus E, *et al.* Improvements to the APBS biomolecular solvation software suite. *Protein Sci* **27**, 112-128 (2018).

5. PYMOL. *The PyMOL Molecular Graphics System, Version 2.0.6 Schrödinger, LLC*
